# Genome-wide analysis of DNA methylation identifies S100A13 as an epigenetic biomarker in individuals with chronic (≥ 30 years) type 2 diabetes without diabetic retinopathy

**DOI:** 10.1186/s13148-020-00871-z

**Published:** 2020-06-03

**Authors:** Tao Li, Yi Xu, Yongyong Shi, Jianhua Chen, Senlin Lin, Jianfeng Zhu, Xian Xu, Lina Lu, Haidong Zou

**Affiliations:** 1 Shanghai Eye Diseases Prevention & Treatment Center/Shanghai Eye Hospital, No. 380, Kangding Road, Shanghai, 200040 China; 2 Department of Ophthalmology, Shanghai General Hospital, Shanghai Jiaotong University School of Medicine, Shanghai, China; 3 Bio-X Institutes, Key Laboratory for the Genetics of Developmental and Neuropsychiatric Disorders (Ministry of Education), the Collaborative Innovation Center for Brain Science, Shanghai Jiaotong University, Shanghai, China; 4 Shanghai Key Laboratory of Psychotic Disorders, Shanghai Mental Health Center, Shanghai Jiaotong University School of Medicine, Shanghai, China

**Keywords:** Diabetic retinopathy, T2DM, Biomarkers, DNA methylation, S100A13

## Abstract

**Background:**

This study aimed to determine the epigenetic biomarkers of diabetic retinopathy (DR) in subjects with type 2 diabetes mellitus (T2DM). This retrospective study is based on the Shanghai Xinjing community prevention and treatment administrative system of chronic diseases. The subjects enrolled herein were T2DM patients who had undergone long-term follow-up evaluation in the system. Two consecutive studies were conducted. In the discovery cohort, among 19 subjects who had developed DR with a DM duration < 3 years and 21 subjects without DR > 30 years after being diagnosed with DM, an Infinium Human Methylation 850 Beadchip was used to identify differential methylation regions (DMRs) and differential methylation sites (DMSs). The function of the genes was assessed through KEGG enrichment analysis, Gene Ontology (GO) analysis, and pathway network analysis. In the replication cohort, 87 DR patients with a short DM duration and 89 patients without DR over a DM duration > 20 years were compared to assess the association between DMSs and DR upon pyrosequencing.

**Results:**

A total of 34 DMRs were identified. Genes containing DMSs with the top 5 highest beta value differences between DR and non-DR participants were located on chromosome 1 and were present in the S100A13 gene, which was associated with 71 GO terms. Two S100A13 gene sites, i.e., cg02873163 and cg11343894, displayed a good correlation with DR on pyrosequencing.

**Conclusions:**

DMSs in the S100A13 gene may be potential biomarkers of DR.

## Background

Epigenetic determinants reportedly contribute to the etiology of numerous systemic disorders such as diabetes mellitus (DM) [1, 2]. Furthermore, hyperglycemia can affect DNA methylase activity or the methylation level of specific genes, which is the primary epigenetic modification [3–7]. Diabetic retinopathy (DR) is a common complication of DM and a leading cause of blindness among individuals aged 20–62 years in developed countries [8]. However, the exact etiology of DR remains unclear. Chen et al. reported that approximately 300 differential methylation sites (DMSs) in DNA were associated with DR onset in type 1 DM (T1DM) patients [9]. Agardh et al. reported an association between DNA methylation and proliferative DR among individuals with T1DM [5]. However, among individuals with T2DM, no specific gene methylation levels have been reported to be associated with DR, even in PubMed.

In the present study, we assessed the epigenetic biomarkers of DR in T2DM patients. In the discovery cohort, among 19 subjects who developed DR with a DM duration of < 3 years and 21 subjects without DR > 30 years after being diagnosed with DM, > 850,000 gene sites were assessed with a methylation chip. The functions of genes harboring DMSs with different degrees of methylation between the DR and non-DR participants were assessed through KEGG enrichment analysis, Gene Ontology (GO) analysis, and pathway network analysis and assessed for their association with DR. In the replication cohort, 87 DR patients with a short DM duration and 89 patients without DR over a DM duration > 20 years were compared to validate the association between the DMSs identified in the discovery cohort and DR through pyrosequencing.

## Results

### Discovery study

The basic and clinical characteristics of the 19 DR and 21 non-DR subjects are summarized in Table 1. Except for differences in the duration and hemoglobin A1C (HbA1c) levels, the two groups did not significantly differ in age, sex, glucose levels, body mass index (BMI), spherical equivalent rate, intraocular pressure, or axial length. In the DR group, one had proliferative DR, 5 patients had severe nonproliferative DR, and 13 patients had moderate nonproliferative DR. Diabetic macular edema was detected in 3 patients. The 21 non-DR subjects did not present signs of DR on annual eye examination after they were diagnosed with T2DM. The duration of DM among these subjects was > 30 years.

**Table 1:** The basic and clinical characteristics of the 19 diabetic retinopathy patients (type 2 diabetes mellitus duration of < 3 years) and 21 patients with no sign of diabetic retinopathy with a type 2 diabetes mellitus duration of > 30 years enrolled in the discovery study

Characteristics*	DR group	Non-DR group	*P* value^#^
Number	19	21	/
Sex (male)	10	10	1
Age (year)	68.74 ± 8.69	74.19 ± 8.59	0.05
Age of DM onset (year)	67.26 ± 8.59	40.10 ± 9.13	0.00
DM duration (year)	1.47 ± 1.07	34.09 ± 5.74	/
Blood glucose (mmol/L)	6.95 ± 1.94	6.99 ± 2.22	0.93
HbA1c (%)	6.79 ± 1.26	7.53 ± 1.44	0.04
BMI (kg/m^2^)	25.30 ± 4.55	24.94 ± 3.33	0.94
MAP (mmHg)	102.98 ± 12.53	103.37 ± 13.45	0.93
SER (D)	− 0.53 ± 2.06	0.17 ± 2.01	0.24
IOP (mmHg)	13.80 ± 4.24	15.02 ± 2.62	0.28
AL (mm)	23.23 ± 1.13	23.26 ± 0.86	0.93

*DR* diabetic retinopathy, *T2DM* type 2 diabetes mellitus

*BMI body mass index; *MAP* (mmHg) mean arterial pressure, equal to (systolic pressure + 2*diastolic pressure)/3; *SER (D)* spherical equivalent rate, equal to spherical power + 1/2 cylindrical power; IOP: intraocular pressure; AL: axial length.

^#^*X*^*2*^ Chi-square test, *t* Student’s *t* test, *Z* Mann-Whitney test

We identified thirty-four differential methylation regions (DMRs) when comparing the DR group and non-DR group. The DMRs comprised 11 hypermethylated regions and 23 hypomethylated regions. Of the 12 gene-related sites, two DMSs were located at the transcriptional start site (TSS) 1500, one at TSS 200, one in the 5′-untranslated region UTR-5, three in EXON1, two in the gene body, and three in the 3′-UTR (Table 2).

**Table 2:** Differential methylation regions corresponding genes and *β* values of diabetic retinopathy and nondiabetic retinopathy subjects in the discovery study

DMR	Gene	***β*** value			*P* value
		DR group	Non-DR group	Difference	
TSS1500	MDFI	0.414024	0.554308	− 0.140284	0.000962
	PGBD5	0.538043	0.365315	0.17273	0.000358
TSS200	ADRA2C	0.042839	0.27238	− 0.229541	0.002349
UTR5	CNTNAP5	0.256273	0.445609	− 0.189336	0.012985
EXON1	PRSS23	0.027501	0.168661	− 0.14116	0.018082
	S100A13	0.509213	0.660521	− 0.151308	1.1E−05
	TIGD5	0.112166	0.27952	− 0.167354	0.001071
GENE BODY	COQ3	0.463529	0.637303	− 0.173774	0.021585
	MOSC2	0.451381	0.634923	− 0.183542	0.00509
UTR3	FLVCR1	0.442131	0.619624	− 0.177493	0.048598
	HLA-B	0.088913	0.308803	− 0.21989	0.001479
	RRH	0.800984	0.629198	0.17179	0.003695
ISLAND	chr1:19110720-19110932	0.56494	0.708782	− 0.143842	0.038852
	chr15:22982192-22982410	0.93844	0.771584	0.16686	0.035381
NSHELF	chr12:120933649-120934713	0.376657	0.609267	− 0.23261	0.026168
	chr14:105603310-105603532	0.730847	0.544063	0.18678	0.03281
	chr17:35165323-35165983	0.636466	0.394896	0.24157	0.009788
	chr18:21083161-21084153	0.890993	0.690135	0.20086	0.008755
	chr4:4119800-4120035	0.5605	0.294444	0.26606	0.017018
	chr4:47427692-47427905	0.54905	0.703312	− 0.154262	0.04677
	chr4:81951941-81952808	0.65228	0.810351	− 0.158072	0.026402
NSHORE	chr5:178565716-178565918	0.721065	0.861094	− 0.140029	0.008616
SSHELF	chr1:219786334-219786562	0.218291	0.432222	− 0.213931	0.00867
	chr13:26586287-26586761	0.603417	0.764495	− 0.161078	0.039872
	chr14:71108008-71109332	0.876179	0.589911	0.28627	0.00262
	chr16:25268786-25269728	0.878063	0.59304	0.28502	0.001536
	chr1:6761295-6762131	0.719637	0.559159	0.16048	0.011819
	chr16:85305690-85305892	0.365183	0.543685	− 0.178502	0.045519
	chr22:20459267-20461473	0.495691	0.667066	− 0.171375	0.037325
	chr2:8825106-8826188	0.724995	0.875096	− 0.1501	0.025348
	chr4:120987617-120988156	0.859063	0.656103	0.20296	0.000565
SSHORE	chr10:54631211-54631426	0.480326	0.686335	− 0.206009	0.006475
	chr1:19110720-19110932	0.547506	0.705954	− 0.158447	0.002265
	chr6:169195967-169196186	0.577833	0.762515	− 0.184682	0.010715

*DMR* differentially methylated region, *DR* diabetic retinopathy, *TSS* transcriptional start site, *UTR* untranslated region, *MDFI* MyoD family inhibitor, *PGBD5* piggyBac transposable element derived 5, *ADRA2C*adrenoceptor alpha 2C, *CNTNAP5* contactin-associated protein-like 5, *PRSS23*serine protease 23, *S100A13*calcium-binding protein A13, *TIGD5*tigger transposable element derived 5, *COQ3*coenzyme Q3 methyltransferase, *MOSC2*mitochondrial amidoxime-reducing component 2, *FLVCR1*feline leukemia virus sub-group C cellular receptor 1, *HLA-B*major histocompatibility complex, class I B, *RRH* retinal pigment epithelium-derived rhodopsin homolog

**Fig. 1:**
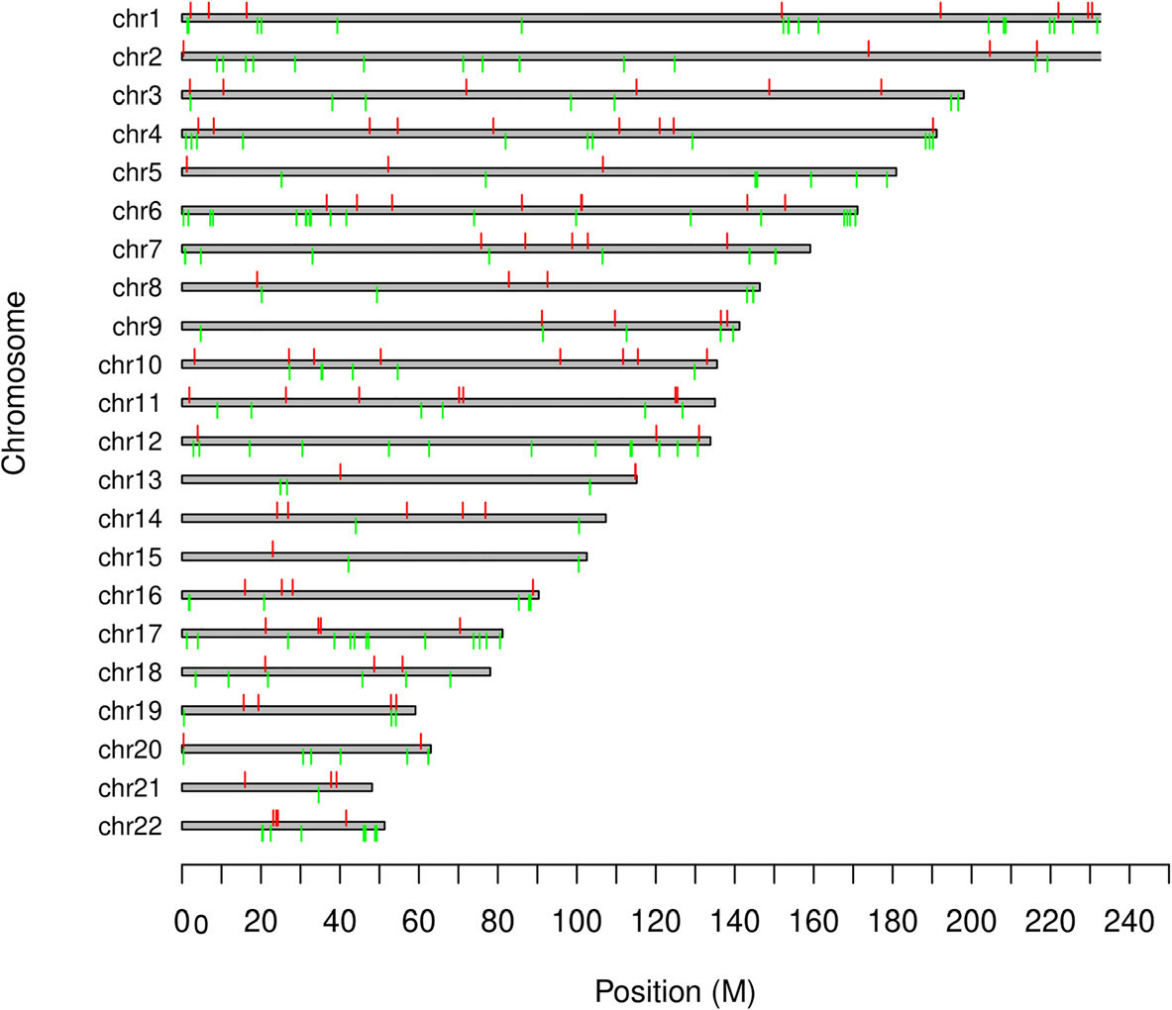
The distribution of differential methylation sites (DMSs). Red markers represent the hypermethylation sites of the DR group, and green markers represent the hypomethylation site

Global DNA methylation levels did not significantly differ between the DR group and the non-DR group (*P* > 0.05). Furthermore, among 290 DMSs, methylation levels were decreased at 188 sites and increased at the other 102 sites in the DR group (Supplement 1). These DMSs were evenly distributed on all chromosomes (Fig. 1). The genes containing DMSs with the top 5 differences in beta values between DR and non-DR participants are listed in Table 3; these DMSs were detected in the S100A13 gene.

**Table 3:** Genes harboring differential methylation sites (DMSs) with the top 5 differences in *β* values between diabetic retinopathy (DR) and non-DR subjects in the discovery study

DMS	Gene	C	Location	*β* value			*P* value
				DR group	Non-DR group	Difference	
cg11596404	S100A13	1	TSS200; 5′ UTR; TSS1500;	0.530201	0.679582	− 0.149381	6.39E−07
cg02873163	S100A13	1	TSS200; 5′ UTR; 5′ UTR; TSS1500	0.550267	0.706868	− 0.156601	6.42E−06
cg17776284	S100A13	1	5′ UTR; TSS200; 1stExon; TSS1500	0.475831	0.621	− 0.145169	1.51E−05
cg11915664	S100A13	1	TSS200; 5′ UTR; TSS1500	0.410099	0.552284	− 0.142185	1.55E−05
cg11343894	S100A13	1	5′ UTR; 1stExon; TSS200;TSS150;	0.504602	0.658721	− 0.154119	1.78E−05

*DMS* differentially methylated site, *DR* diabetic retinopathy, *C* chromosome, *S100A13*calcium-binding protein A13, *TSS* transcriptional start site, *UTR* untranslated region

**Fig. 2:**
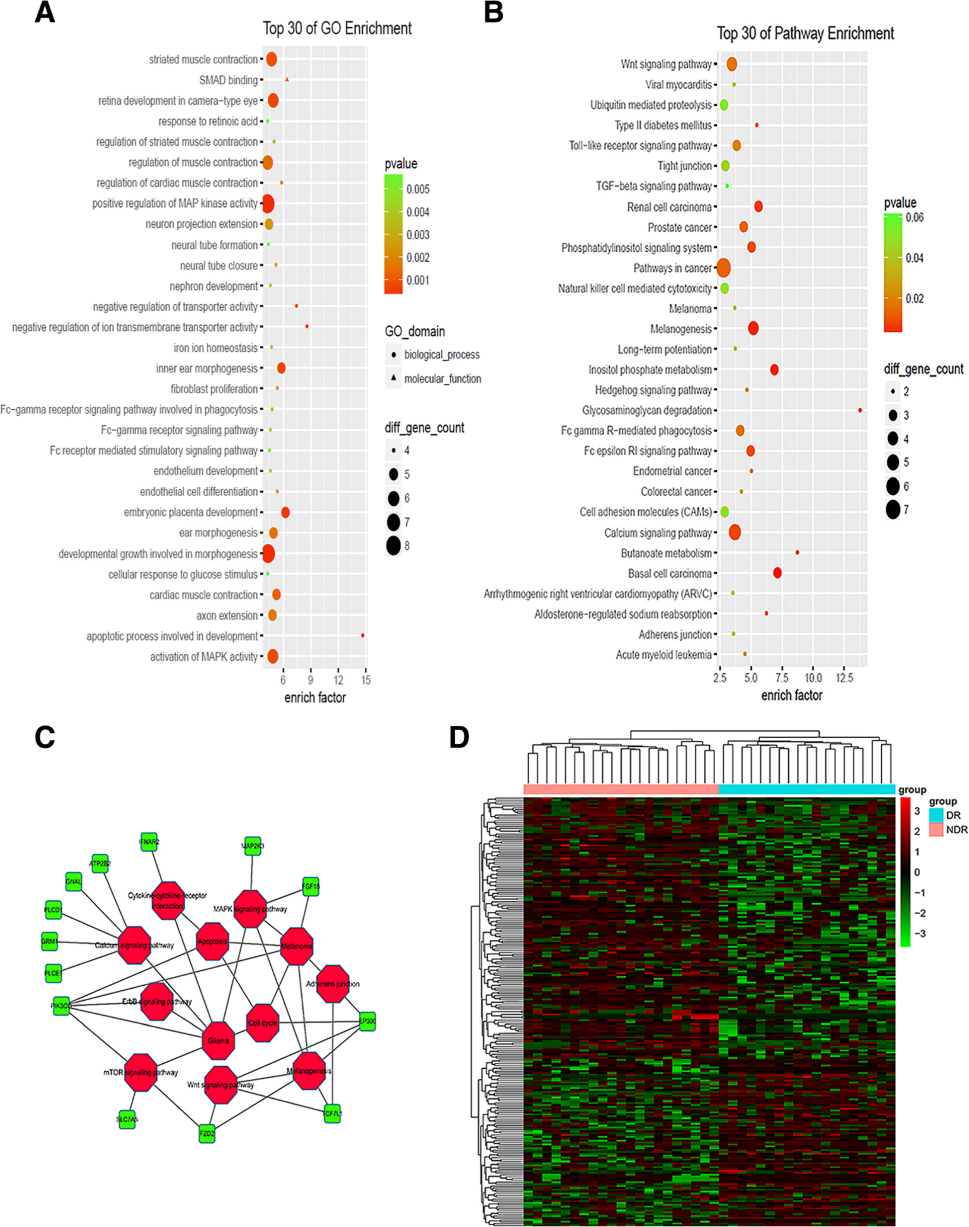
The GO enrichment map of differential methylation sites (DMSs), and the top 30 items are displayed in the graph. The larger the enrichment value is, the more significant the enrichment is. Fig. 2**b** shows the KEGG pathway analysis of DMSs in genes, and the top 30 items are displayed in the graph. Figure 2**c** shows the potentially most important pathway network, and the differentially methylated genes are involved in pathways of cytokine receptor signaling, MAPK signaling pathway, apoptosis, calcium signaling pathway, adhesion factor, ErbB signaling pathway, cell cycle, Wnt signaling pathway, mTOR pathway, and others. Figure 2**d** shows the heat map of DMS in the diabetic retinopathy (DR) group comparing to the non-DR group. The sites are classified to one of the following eight regions: TSS1500 (1500 bp upstream to 200 bp upstream of TSS), TSS200 (200 bp upstream of TSS), 5′ UTR, the first exon (1st Exon), 3′ UTR, other exon (excluding the first exon), and intronic and intergenic regions. Red indicates DMSs above the average level, and green indicates DMSs below the average of all of the samples

On GO enrichment analysis and KEGG pathway analysis (Supplement 2 and 3, respectively), the top 30 items are summarized in Fig. 2 a and b, respectively. DR-associated pathways included the Wnt signaling pathway, ubiquitin-mediated proteolysis, Toll-like receptor signaling pathway, tight junctions, the transforming growth factor-beta signaling pathway, the phosphatidylinositol signaling pathway, natural killer cell-mediated cytotoxicity, inositol phosphorylation metabolism, the hedgehog signaling pathway, aminoglycan degradation, Fcγ receptor-mediated autophagy, cell adhesion factor signaling, the calcium signaling pathway, regulation of muscle contraction, neural tube formation, neural cell development, iron ion balance, fibroblast proliferation, phagocytosis associated with Fcγ receptor pathogenesis, glucose-stimulated cellular responses, and developmental processes. Differential methylation in apoptotic responses and genes involved in AMPK activity are indicated in Fig. 2 a, b, and c. S100A13 was associated with 71 GO terms, including signal transduction and calcium ion binding, which are reportedly associated with DR pathogenesis [10, 11]. Therefore, the DMSs of the S100A13 gene were considered candidate DMSs.

### Replication study

The basic and clinical characteristics of the 87 DR and 89 non-DR subjects are summarized in Table 4. Except for DM duration and HbA1C levels, no significant differences were observed in blood glucose levels, BMI, intraocular pressure, equivalent spherical rate, and axial length between the two groups. The 89 participants in the non-DR group did not present signs of DR on annual eye examination after being diagnosed with T2DM. Their disease durations were > 20 years.

**Table 4:** Basic and clinical characteristics of the 87 diabetic retinopathy patients (type 2 diabetes mellitus duration of < 3 years) and the 89 patients with no sign of diabetic retinopathy with a type 2 diabetes mellitus duration of > 20 years enrolled in the replication study

Characteristics*	DR group	Non-DR group	*P* value
Number	87	89	/
Sex (male)	38	34	0.54
Age (year)	66.55 ± 8.27	74.60 ± 8.80	0.00
Age at DM onset (year)	64.45 ± 8.12	48.40 ± 9.17	0.00
DM duration (year)	2.10 ± 0.95	26.19 ± 4.17	0.00
Blood glucose (mmol/L)	7.48 ± 2.44	7.93 ± 2.51	0.22
HbA1c (%)	7.02 ± 1.37	7.83 ± 1.36	0.00
BMI (kg/m^2^)	25.31 ± 3.98	25.95 ± 7.03	0.46
MAP(mmHg)	101.65 ± 14.23	96.61 ± 14.06	0.35
SER (D)	0.04 ± 2.14	− 0.17 ± 2.22	0.67
IOP (mmHg)	13.72 ± 3.51	14.08 ± 3.83	0.53
AL (mm)	22.52 ± 4.44	22.63 ± 4.65	0.30

*DR* diabetic retinopathy, *T2DM* type 2 diabetes mellitus

*BMI body mass index; *MAP (mmHg)* mean arterial pressure, equal to (systolic pressure+ 2*diastolic pressure)/3; *SER (D)* spherical equivalent rate, equal to spherical power + 1/2 cylindrical power; *IOP* intraocular pressure; *AL* axial length

The association between DR and DMS in the S100A13 gene was verified through pyrosequencing (primer sequences are provided in Supplement 4). Significant differences (*P* < 0.05) between the DR and non-DR groups were observed at sites cg02873163+12 (41.91 ± 12.60%; 47.73 ± 12.36%), cg02873163+2 (54.99 ± 14.77%; 61.45 ± 13.68%), cg02873163 (51.03 ± 14.63%; 57.21 ± 13.26%), cg11343894+5 (45.86 ± 15.88%; 51.47 ± 14.34), and cg11343894 (48.83 ± 15.17%; 54.51 ± 14.45) in the S100A13 gene. After adjusting for age, sex, BMI, HbA1C, and mean arterial pressure, the average methylation level of all 5 sites of cg02873163 and cg11343894 was positively associated with DR through binomial logistic regression analysis (Table 5).

**Table 5:** Binomial logistic regression analysis of the associations between diabetic retinopathy and methylation levels (%) at the differential methylation sites

	Beta (95%CI)	*P*
cg02873163+12(%)	1.039(1.007,1.072)	0.017
cg02873163+2(%)	1.033(1.005,1.061)	0.020
cg02873163 (%)	1.034(1.006,1.063)	0.019
cg11343894+5(%)	1.025(1.000,1.051)	0.048
cg11343894(%)	1.026(1.000,1.053)	0.046

## Discussion

To identify possible epigenetic biomarkers of DR among individuals with T2DM, we enrolled T2DM patients segregated into a discovery cohort and a replication cohort and identified 34 DMRs associated with DR in Chinese individuals with T2DM. These 34 DMRs included 23 (67.65%) hypomethylation sites, concurrent with a previous report on T1DM patients [5]. DR results from a cascade of enzymatic reactions. With the regulation of DNA methyltransferases and ten-eleven-translocation proteins, DNA hypermethylation/demethylation alters the binding of transcription factors and genes in most cases [12–14]. Interactions between genetic and epigenetic factors may play a role in DR pathogenesis.

Chen et al. reported that different blood glucose levels affect the extent of DNA methylation in specific genes in whole blood or monocytes, which subsequently affects DR pathogenesis [9]. Herein, blood glucose and HbA1c levels were lower among DR subjects than among non-DR subjects, indicating that the etiology of DR is complex and does not simply depend on glucose levels. We show that the S100A13 gene is potentially involved in DR pathogenesis and that the cg02873163 and cg11343894 sites in the S100A13 gene may serve as potential biomarkers of DR.

The S100A13 gene is involved in calcium ion homeostasis, energy metabolism, inflammation, apoptosis, regulation of proliferation, differentiation, and interactions with transcription factors and nucleic acids within cells and activates surface receptors, including the receptor for advanced glycation end-products and Toll-like receptor 4, G-protein-coupled receptors, scavenger receptors, or heparan sulfate proteoglycans and N-glycans [15, 16]. Furthermore, S100A13 regulates calcium levels and fibroblast growth factor-1 and interleukin-1 alpha secretion through a noncanonical pathway [10, 11, 17]. Mandinov et al. reported that S100A13 mediates IL-6-induced damage to the macrovasculature [18]. We speculate that significant demethylation of S100A13 in individuals with DR downregulates S100A13 and upregulates p38 MAPK and nuclear factor-kappa B through calcium signaling and the RAGE pathway, thus increasing the damage caused by hyperglycemia [17, 19–23]. Patients harboring hypomethylated S100A13 may require closer follow-up evaluation and more stringent blood glucose screening.

It is generally difficult to confirm a gene-negative contrast group in genetic studies on chronic diseases. For example, DR may occur throughout the lifetime of an individual with T2DM, and the exact diagnosis of non-DR can only be made towards the end of an individual’s lifespan. Herein, we selected non-DR individuals with a maximal DM duration to serve as non-DR participants. Furthermore, we selected individuals who developed DR with a DM duration of < 3 years as the gene-positive group, since we believe that epigenetic changes initiate retinal neural and vascular impairment within a short period. This study cohort displayed large differences in age, onset age, and duration of DM between the two groups, subsequently leading us to speculate whether differences in DNA methylation are associated with age or duration of T2DM. Therefore, we conducted two consecutive studies, including different non-DR contrast groups with different DM durations, and found that DMSs in the S100A13 gene were present in both studies; moreover, the associations between DMSs and DR were confirmed with regression analysis.

This study has several limitations. First, subjects developing DR within a relatively short duration or those without DR in the long term may harbor gene mutations, which potentially affect approximately 14% of the methylation levels at corresponding or adjacent sites [24]. Second, each cell type has a different DNA methylation programming system [18]. DMSs in whole blood cells can only reflect the potential for the occurrence of diabetic vascular disease instead of duplicate DMSs in retinal cells. Finally, 22 DMRs are present in CpG islands located distal to any known gene among the 34 DMRs, and their functions warrant further elucidation in future studies.

## Conclusions

Our study is the first to detect DMRs and DMSs associated with DR in T2DM patients, indicating that DMSs in the S100A13 gene serve as potential biomarkers of DR. Future studies with more genetically diverse cohorts having a shorter DM duration of < 10 years and prospective cohort studies are required to confirm the effect of epigenetic changes in the S100A13 gene on DR incidence.

## Methods

This retrospective study is based on the Shanghai Xinjing community prevention and treatment system of chronic diseases, which was established in 1999. The system has curated annually updated health data on all local residents diagnosed with T2DM. In 2003, our group participated in this system, and we have been conducting and supervising studies on the prevention and treatment of ocular diseases [25–29]. From June to August 2016, we conducted an epidemiological study on eye complications of DM upon receiving ethical approval by the Ethics Committee of Shanghai General Hospital, Shanghai Jiaotong University School of Medicine (2013KY023). This study describes the epigenetic analysis conducted within that study. This study adheres to the tenets of the Helsinki Declaration, and the study subjects provided written informed consent to participate in the study.

The inclusion criteria were as follows: (1) Chinese Han background, (2) a diagnosis of T2DM in accordance with the World Health Organization criteria [30], and (3) the ability to comply with all the required examinations. The exclusion criteria were as follows: (1) occurrence of eyelid diseases, strabismus, corneal diseases, lens diseases, and other eye diseases potentially affecting the outcomes of retinal examinations; (2) occurrence of other eye diseases, including glaucoma or macular degeneration, which may cause fundus retinal microvasculopathy; (3) history of eye surgery or trauma; (4) occurrence of severe systemic diseases, including those involving the respiratory system, circulatory system, and excretory system; and (5) history of cancer.

The research staff were fully trained and experienced with epidemiologic studies [25–27]. We gathered data including demographic characteristics and conducted a routine eye examination. DR was diagnosed on the basis of the well-accepted international diagnostic criteria. For each participant, 2 mL samples of fasting blood were obtained, the cell composition was determined for further data analysis, and DNA was extracted using a QIAN amp blood kit (Qiagen, Hilden, Germany) in accordance with the manufacturer’s instructions.

### Discovery study

#### Enrolled participants

The discovery cohort included 19 subjects who developed DR with a DM duration of < 3 years and 21 subjects without DR > 30 years after being diagnosed with DM.

#### DNA methylation quantification

The genomic DNA (500 ng) was subjected to bisulfite conversion using an EZ DNA methylation kit (Zymo Research, Orange, CA, USA) in accordance with the manufacturer’s instructions. An Infinium Human Methylation 850 Beadchip methylation chip (Illumina, San Diego, CA, USA), which covers 99% of all RefSeq genes and contains 867,531 sites, was used. Methylation analysis involved data quality control, preprocessing, methylation difference site analysis, and methylation difference region analysis.

The quality control step for the analysis of the 850k chip included checking for sex (X,Y-chromosome data were not included in the DMS/DMR analysis) [31], filtering the SNP sites (minor allele frequency > 5% and probes with SNPs of minor allele frequency > 5% within 10 base pairs of the CpG sites were excluded) [32], (list of CpG sites is available at http://genetics. emory.edu/research/conneely/annotation-r-code.html), normalization (Subset-quantile Within Array Normalization was used to normalize the DNA methylation probe intensity) [33], following the criteria for retaining probes (detection *P* < 0.05 in more than half of the samples) and individuals (detection *P* value of more than 95% of the probes of each sample < 0.05).

### Replication study

#### Enrolled participants

Pyrosequencing was performed to verify the correlation between the candidate DMSs and DR. The replication cohort included 87 subjects who developed DR with a DM duration of < 3 years and 89 subjects without DR over a DM duration > 20 years.

#### Pyrosequencing

DNA (500 ng) was subjected to bisulfite conversion with the EZ DNA methylation kit (Zymo Research) in accordance with the manufacturer’s instructions. Pyrosequencing was performed with the PyroMark Q96 ID system (Qiagen, Valencia, CA, USA) in accordance with the manufacturer’s recommendations. The PyroMark PCR Master Mix kit (Qiagen), streptavidin-coated beads (GE Healthcare, Uppsala, Sweden), PyroMark Gold Q96 reagents (Qiagen), PyroMark Q96 Vacuum Workstation, and PyroMark Q96 software (version 2.5.8, Qiagen) were used to determine and analyze DMSs.

#### Data processing and statistical analysis

The clinical data were statistically analyzed using the SPSS 22.0 software (IBM Corporation, Armonk, NY, USA). The data are expressed as the mean ± standard deviation values, and calculated values are presented as frequencies and percentages. Data normality was assessed using the Kolmogorov-Smirnov test. Normally distributed data were analyzed using Student’s *t*-test to compare differences in various values between the DR and non-DR groups. Skewed data were analyzed using the Mann-Whitney test. Statistical tests performed herein were the chi-square test and independent samples *t* test.

Minfi software package (version 1.25.1, http://www.bioconductor.org/packages/release/bioc/html/minfi.html) and IMA software package (version 2.0, http://www.rforge.net/IMA), both written with R software, were used to determine the differences in the degree of DNA methylation between the DR participants and the non-DR participants [34]. Minfi package was used to calculate the beta value (interval between 0 and 1), an indicator of the degree of methylation using the following equation: ${\mathrm{beta}}_{\mathrm{i}}=\frac{\max \left({y}_{\left(\mathrm{i},\mathrm{methy}\right)},0\right)}{\max \left({y}_{\left(\mathrm{i},\mathrm{methy}\right)},0\right)+\max \left({y}_{\left(\mathrm{i},\mathrm{unmethy}\right)},0\right)+100}$. The distribution of the beta values was skewed, and all outlier beta values were excluded (omitted values > 4 SD).

The sitetest algorithm and the regionswrapper algorithm, both from the IMA package, were used to define DMS and DMR, respectively. Du et al. suggested the threshold of beta value as between 0.05 and 0.15 [35], and according to the Illumina’s instructions, 0.14 is the minimum value recommended to guarantee the proper sensitivity. Therefore, if differences between beta values at a certain region or site between the DR and non-DR groups were greater than 0.14 and the *P* value determined via a pooled *t* test was less than 0.05, the region was considered a DMR, and the site was considered a DMS without regression analysis.

The functions of genes containing DMRs or DMSs were assessed via KEGG enrichment analysis, GO analysis, and pathway network analysis. We mapped the methylated sites to GO terms in the Gene Ontology database and determined and corrected (Bonferroni) the number of genes for each GO term. KEGG pathway analysis (http://www.kegg.jp/) was performed to confirm the enriched pathways. The threshold for statistical significance (< 0.05) was defined on the basis of the corrected *P* value. The gene coexpression network was constructed in accordance with the normalized signal intensity to identify gene interactions. A strong correlation (Pearson correlation > 0.9) indicated the interaction between two genes. Cytoscape (National Institute of General Medical Sciences, Boston, MA, USA) was used to construct coexpression networks. If a gene harbored a DMS with higher differences in beta values between DR and non-DR participants and the corresponding protein was involved in previously reported pathways associated with DR pathogenesis, the DMS was considered a candidate DMS in the following replication study. The R 3.5.1 pheatmap package was used to calculate and draw the heat map.

In the replication part, the association between methylation level (%) on pyrosequencing and DR was adjusted for demographic characteristics, including age, sex, Hb1Ac (%), body mass index, and mean arterial pressure, which were assessed through binomial logistic regression analysis. A *P* value < 0.05 was considered statistically significant.

## Supplementary information

**Additional file 1:.** Supplement 1.

**Additional file 2:.** Supplement 2.

**Additional file 3:.** Supplement 3.

**Additional file 4:.** Supplement 4.

## Data Availability

The datasets used and/or analyzed during the current study are available from the corresponding author on reasonable request.

## Abbreviations

DR: Diabetic retinopathy
T2DM: Type 2 diabetes mellitus
DMG: Differential methylation region
DMS: Differential methylation site
GO: Gene Ontology
S100A13: Calcium-binding protein A13
T1DM: Type 1 DM
HbA1c: Hemoglobin A1C
BMI: Body mass index
MAP: Mean arterial pressure
SER: Spherical equivalent rate
IOP: Intraocular pressure
AL: Axial length
TSS: Transcription start site
UTR: Untranslated region
MDFI: MyoD family inhibitor
PGBD5: PiggyBac transposable element derived 5
ADRA2C: Adrenoceptor alpha 2C
CNTNAP5: Contactin-associated protein-like 5
PRSS23: Serine protease 23
S100A13: Calcium-binding protein A13
TIGD5: Tigger transposable element derived 5
COQ3: Coenzyme Q3 methyltransferase
MOSC2: Mitochondrial amidoxime reducing component 2
FLVCR1: Feline leukemia virus sub-group C cellular receptor 1
HLA-B: Major histocompatibility complex, class I B
RRH: Retinal pigment epithelium-derived rhodopsin homolog
C: Chromosome
