# Supplementary material for: Genome-wide analysis of DNA methylation identifies S100A13 as an epigenetic biomarker in individuals with chronic (≥ 30 years) type 2 diabetes without diabetic retinopathy
Source: Clin Epigenetics. 2020 Jun 3;12:77. doi: 10.1186/s13148-020-00871-z (PMC7268721; doi:10.1186/s13148-020-00871-z)

**Supplement 4**

1 cg17094014

>hg19_wgEncodeHaibMethyl450Gm12878SitesRep1_cg17094014 range=chr6:41604843-41605843 5'pad=0 3'pad=0 strand=+ repeatMasking=none

CCCGGCCTGGGGGGCGGAACACTCTCTTCTTGTCCCGCCCCACCTTCCTCCGGGCCGCAACCACCCCAGCCCATCGCTGGGACGTGGCCGCGGGTGCCGAGAGCGCTGCCGGCGCCGTGGGGGCCATCCCGGTCCTGGCCCCGACGGCGCCCCAGGACCTGCGATAGCCGTAAGTAGCGACTATGGGGGCTAGGAGAGTCCAAGAAGTGGGGAGCTGGGACGCCCGGCCTCCTGCGTCCCAGCTCTCCACTTCCAGACCCGGCTGGACTCGGGACTAGAAAAGGTGGCCTGGTGCCGCCGCTGCCTTCCAGTGTGCTCACTGGCCCTCGTGGCGGGGAGCCCAGACGCGGCTCCTAGGCGGCCCTAGACAAGTACGGCCGAGACGCTGTCACCCCAGGGGGCTGCCCCACCTGAGCATCATCCCACGGGCCCGCCCAGCCCCACCTGGAGAGCCGCAGGCTCCAAAGTCTTCCCCATTCTCGGGGACCAGGTTCCGGGGTCGCGCTGAGGGCCCCTGAACCCTCTTGGACTGCCTGGCAGCGGCTTCTTCCTACCACCACGGCTCACCATCCAGACGCGCCGCCCGGCTGGCCCCACCGGGAGAGGCTCCCTCCCTCCCCTTTCTCCCCGAACCCTCATCTTAGAAGCCCTCGGATTCAGTCTGGATCCCAAAGAAGGGCTGAATTCGGAGGACCTCTCTTTGCCCCCAAAGTCGGTTTTCTGACACACATTCCCACGCACAGCTGGTAGGGGCAGGAGGGTCGAGGATGCTCTGGGCTGCTGCGGCACAGGGCAGGCTGGTTGGTCACTAACCGGATTTCCCGGACAGACAACTCTGGCGCTGTGGGCGGGTGGCCTGGGAGCCCAGATCCCTGTCCCTGGCTTAGGACCCCGGGAGGAGACCGAGGGGGTGGGTAAAACGTGACTGAGAAACTTGATCGCGCCCATCTTTGGGGCAAAGAGATTTGGAGGCCGCAATTATTCAGACAGTTCCACGAAGT

Reverse sequence design primer：（pos2）

| **Primer Set 1** | | | | **Score: 82 Quality: Medium** | | |  |
| --- | --- | --- | --- | --- | --- | --- | --- |
| **Primer** | **Id** | | **Sequence** | **Nt** | **Tm, ºC** | **%GC** |  |
| 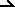PCR | F1 | | AGTAGTGGTGGTAGGAAGAAGT | 22 | 60.0 | 45.5 |  |
| 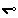PCR | R1 | | CAAACTCCAAAATCTTCCCCATTC | 24 | 60.9 | 41.7 |  |
| 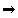Sequencing | S1 | | GTTTAAGAGGGTTTAGGG | 18 | 46.4 | 44.4 |  |
| Target Polymorphisms | Position11, Position12, Position13 | | | | | | |
| Sequence to Analyze | GTTTTTAGYG YGATTTYGGA ATTTGGTTTT | | | | | |  |
| Amplicon length | | 109 | | | | | |


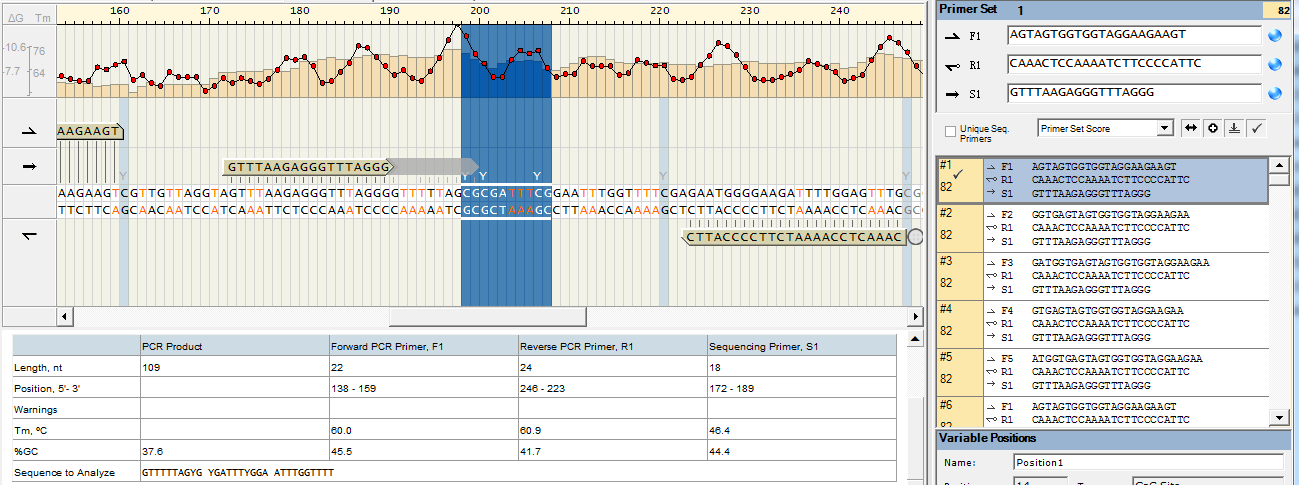


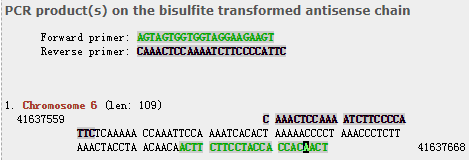


2 cg08477332

>hg19_wgEncodeHaibMethyl450Gm12878SitesRep1_cg08477332 range=chr1:153589744-153590744 5'pad=0 3'pad=0 strand=- repeatMasking=none

AGGGGCAGAGATGTGGGGCAGGAGAGGTGACTCGGGCTCAGCTGCATGGTCCTGCTCTGCTTCTTTTTTCTTGGAGTTTGTCCGTTGGATGACAATGATAGTGGTGAACAGGTATGGAGGGCTTACTAAGTACCAGGTGCAGTGCTAAACATTTTGCCTGTATTGTCTTAGTTAATTCTCACAGTCCTTTGAGGAAGGCACTATTTTTAACTCCTGTATTAAAGATCAGGACACAGATCTTAGTTAAAGTCATAAAGACAAGAATAGGTGGATTTCAGAGCTCTGATTAACCAGGATCCTCTCTGGCTGGGTGCTCAGGAACACTAGGGCCTCCTCCCCTTACCCTCTGGAGCAGTGGGGAAGCCAGAAGGAGGGGCCCGATGTTCTGCTCAAGGACCGTGGGGTGAGGGTGTGGTGGTGCCACCGGAGGCAGAACTTGCCTTGCCTTCCTGGCTTGAGGGGCATATGGTGTACACGGCCACCACCAGAGGGAGCAGGGGCGGGCATCTCGGTGACCAGCCTCCCAGGGCTGTCACTGCCTCACTGGCAGGCATCCATGCAGGGCAGCTATGAGTCCAGAGGAACCTTTAGGCTGAGGGAGCTCCCTGACCCTCCGTTAGAGAGCCCCAGACTGAGACCTCAGAGAGACTCCCAGACTGGGAATCCAAAGGGCCATTGGCAGAGACATTCAAGAGGGGCTCCTGCACTGAAGGGCTCCCTTACCGTGAGAAGCCCTGTGAGGAACCCCCTAGCCTGAGCCTCCTCCTGAGGAACCCAGGCTAAGTCACTCTCAGAGGAACCCACAGGCTGAGAGACCCTCAGAGGGACCTGCAGGTTGAAGCCCTTCAGAGGAACTCTCAGAATGAGACATTGTTGGAAGGTCCTCTCCATTGAGACACACAGGGCCCCAGGTTGAGAAGGTGACATGAGGAGGGATGTAGTGGGGTGTGCTCTGATCTCAGCGTTTAGGAAAGGATCCTGGGAACTTTGGCTCTGGGCCG

Note: G is an SNP, which needs to be avoided.


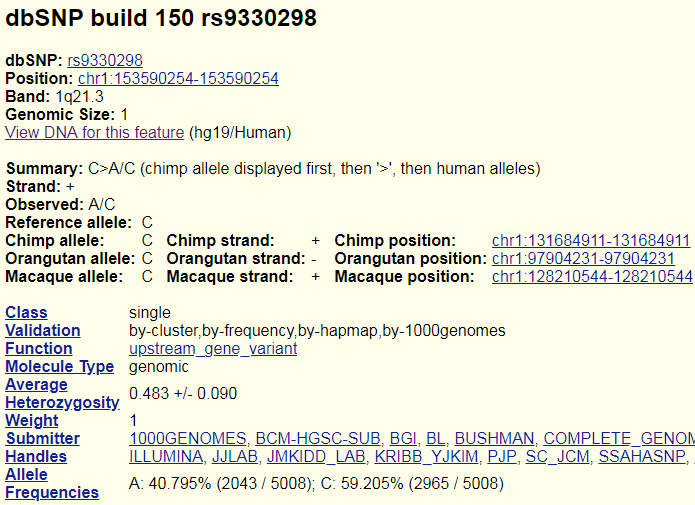


Reverse sequence design primer：（pos2）

| **Primer Set 1** | | | | **Score: 88 Quality: High** | | |  |
| --- | --- | --- | --- | --- | --- | --- | --- |
| **Primer** | **Id** | | **Sequence** | **Nt** | **Tm, ºC** | **%GC** |  |
| 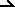PCR | F1 | | TGTTTTGTATGGATGTTTGTTAGTGA | 26 | 58.7 | 30.8 |  |
| 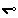PCR | R1 | | ACCTTACCTTCCTAACTTAAAAAACATAT | 29 | 59.5 | 27.6 |  |
| 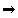Sequencing | S1 | | AGTGATAGTTTTGGGAG | 17 | 45.1 | 41.2 |  |
| Target Polymorphisms | Position5 | | | | | | |
| Sequence to Analyze | GTTGGTTATY GAGATGTTYG TTTTTGTTTT TTTTGGTGGT GGT | | | | | |  |
| Amplicon length | | 128 | | | | | |


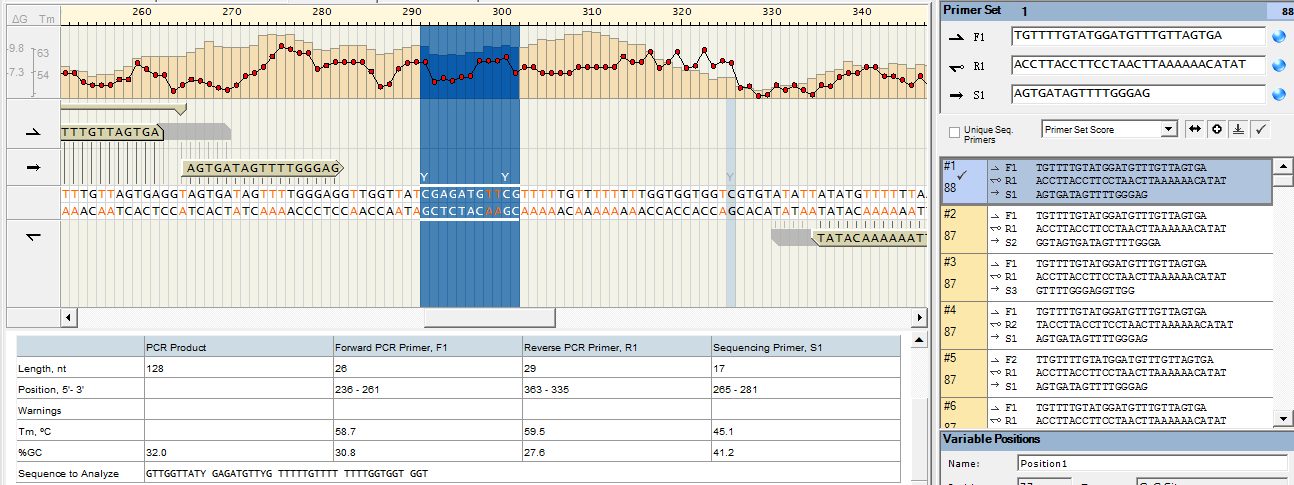

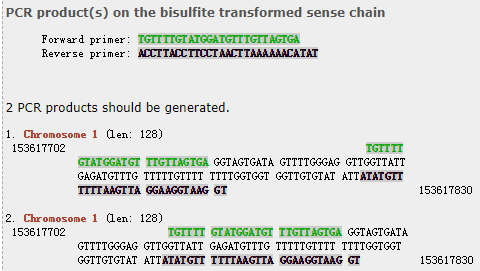


3 cg02873163

>hg19_dna range=chr1:153599329-153600329 5'pad=500 3'pad=500 strand=+ repeatMasking=none

GTCTTCAGTTGAAGGTCCTTCTTGGAGTCCTTCAAGTCTGGTGGCCCCTCTGTGGCGTGTCTGTCTCAAGAATCCCTTCTTGTCTCACCCAGACTCACAGGTCATAAGGTTGTTTGTCTCCAAAGCTTGTTTATGTTGGGCCCTGGTCTCCGGCCACTCGCATCCGCCCACACACACTCTAACCGCTCCTCCCAGCAGAGAAGCACAGGGTCTGGCAAATGTCCCCTGTGGTCAGAGCTGGCTCCGTCTGAACAGACCCATTTTCTCTGCCCCGCTCCACCCTTACCTCTCCTCAGCGGCAGCAGGGCAGAGTGCTGAACCCAGGACCCCACAGATCCTCCCCGCTCCTGTCTCCCGGTGACAAGGGTCCTGGAACGGGGCGTCTCTGACTCCCTGCTCCAGGACGGGTTTAGTACAGGCACTCACAACCCCCTGGGGTGCGGCGGGTGGGGCTGGAAGAGGCATTCTCTTTTCTGTCCACCCCGTCACACACTCCTGCACGCGCTGTTGCTCGATCATCCAAAGCTCCTTCCTGAATTCCTGTCTGAGCTGGCTCTGGGGGAGGGACCTGGGAGCCCAGGAAGAAAAAATGATCTCCAGCCTCCCTCCAGGGTCAGCCATGAGACTCACCCGGCAAGGAGATGGGGTAGAGTGAGCTGGAGCCTCAGGGCTGAGGTTTATAAGCAGCGGGGAAGGAGGAGAGAGCTGCTTCCAAGCCCGGAGGTGTCAAATTTCCGTGGTCGATTCTGAAGACCAGAGACAGCCCAATCAGGCCGCTTGCCCTGAGCCCTCAGGTCTGGACCCGGGCTCCCTGCCAGCCACACGAGCGCCCCCACTTGGTCAGGGAGAGGAGCTGCCTGGACAGATCTCGTTCGGCAGGGGTCCCAGGGTCCTAGTCCTCTCAATACTGGGGGCTGCTGCCACCATACCCATGTCTAATCAGGAGTGTGTATGCTGAGGGGGGAAGAGGGAGGCAACCAATCAAGGGAGCTCAACACTGA

Reverse sequence design primer：（pos3）

| **Primer Set 1** | | | | **Score: 87 Quality: Medium** | | |  |
| --- | --- | --- | --- | --- | --- | --- | --- |
| **Primer** | **Id** | | **Sequence** | **Nt** | **Tm, ºC** | **%GC** |  |
| 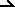PCR | F1 | | TTGGAGGGAGGTTGGAGATT | 20 | 61.2 | 50.0 |  |
| 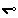PCR | R27 | | TACAAACACTCACAACCCCCTAA | 23 | 60.8 | 43.5 |  |
| 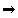Sequencing | S1 | | AAGGAGTTTTGGATGA | 16 | 44.4 | 37.5 |  |
| Target Polymorphisms | Position3, Position4, Position5 | | | | | | |
| Sequence to Analyze | TYGAGTAATA GYGYGTGTAG GAGTGTGTGA | | | | | |  |
| Amplicon length | | 198 | | | | | |


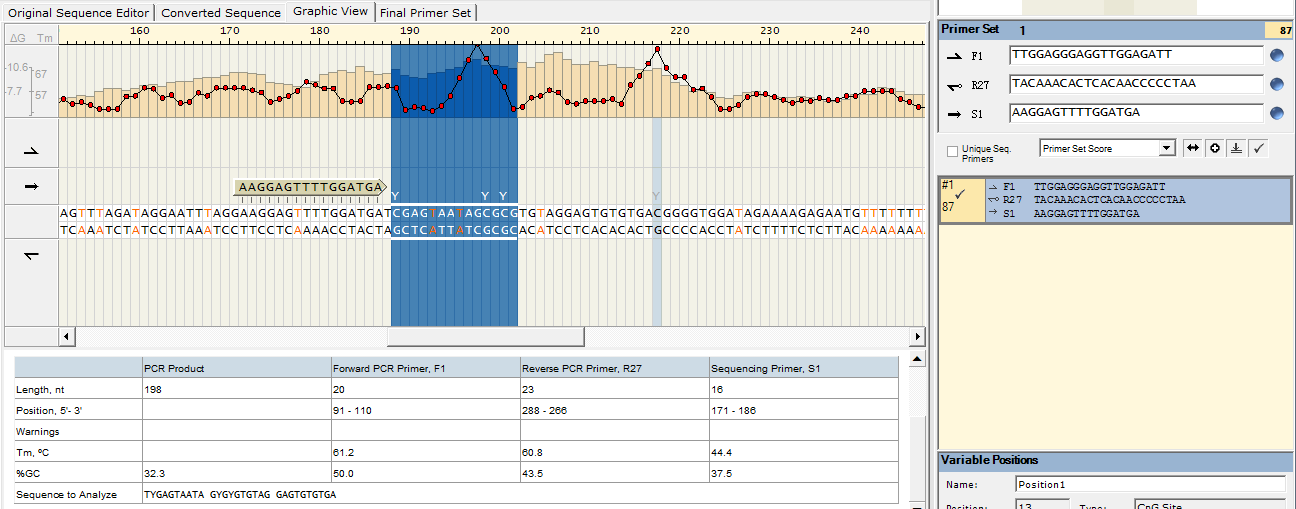


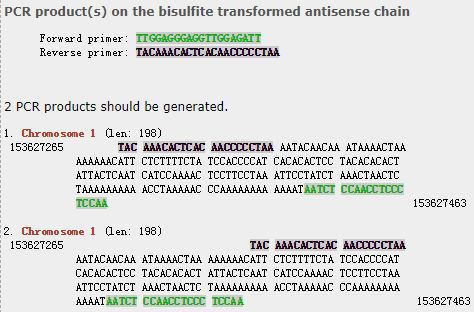


4 cg11343894

>hg19_wgEncodeHaibMethyl450Gm12878SitesRep1_cg11343894 range=chr1:153599204-153600204 5'pad=0 3'pad=0 strand=+ repeatMasking=none

AGATTCAGCTCCAGAAACCCCCTCTTCTGTAGGTCTCACAATCCGGGATCCTTTTTAAATGTCTGTGGCTTAGGGACCCCTCTGTGGTGATCCCTCGATCTGGAGACCCCTCTACTTGAGAGGAGGTCTTCAGTTGAAGGTCCTTCTTGGAGTCCTTCAAGTCTGGTGGCCCCTCTGTGGCGTGTCTGTCTCAAGAATCCCTTCTTGTCTCACCCAGACTCACAGGTCATAAGGTTGTTTGTCTCCAAAGCTTGTTTATGTTGGGCCCTGGTCTCCGGCCACTCGCATCCGCCCACACACACTCTAACCGCTCCTCCCAGCAGAGAAGCACAGGGTCTGGCAAATGTCCCCTGTGGTCAGAGCTGGCTCCGTCTGAACAGACCCATTTTCTCTGCCCCGCTCCACCCTTACCTCTCCTCAGCGGCAGCAGGGCAGAGTGCTGAACCCAGGACCCCACAGATCCTCCCCGCTCCTGTCTCCCGGTGACAAGGGTCCTGGAACGGGGCGTCTCTGACTCCCTGCTCCAGGACGGGTTTAGTACAGGCACTCACAACCCCCTGGGGTGCGGCGGGTGGGGCTGGAAGAGGCATTCTCTTTTCTGTCCACCCCGTCACACACTCCTGCACGCGCTGTTGCTCGATCATCCAAAGCTCCTTCCTGAATTCCTGTCTGAGCTGGCTCTGGGGGAGGGACCTGGGAGCCCAGGAAGAAAAAATGATCTCCAGCCTCCCTCCAGGGTCAGCCATGAGACTCACCCGGCAAGGAGATGGGGTAGAGTGAGCTGGAGCCTCAGGGCTGAGGTTTATAAGCAGCGGGGAAGGAGGAGAGAGCTGCTTCCAAGCCCGGAGGTGTCAAATTTCCGTGGTCGATTCTGAAGACCAGAGACAGCCCAATCAGGCCGCTTGCCCTGAGCCCTCAGGTCTGGACCCGGGCTCCCTGCCAGCCACACGAGCGCCCCCACTTGGTCAGGGAGAGGAGCTGCCTGGACAGATCTCGTTCGG

Reverse sequence design primer：（pos2）

| **Primer Set 1** | | | | **Score: 85 Quality: Medium** | | |  |
| --- | --- | --- | --- | --- | --- | --- | --- |
| **Primer** | **Id** | | **Sequence** | **Nt** | **Tm, ºC** | **%GC** |  |
| 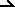PCR | F1 | | TTTTAGGGGGTTGTGAGTGTTTGTAT | 26 | 62.7 | 38.5 |  |
| 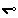PCR | R1 | | CACCCTTACCTCTCCTCAAC | 20 | 59.8 | 55.0 |  |
| 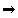Sequencing | S1 | | GGAGTAGGGAGTTAGAG | 17 | 48.0 | 52.9 |  |
| Target Polymorphisms | Position9, Position10 | | | | | | |
| Sequence to Analyze | AYGTTTYGTT TTAGGATTTT TGTTAT | | | | | |  |
| Amplicon length | | 161 | | | | | |


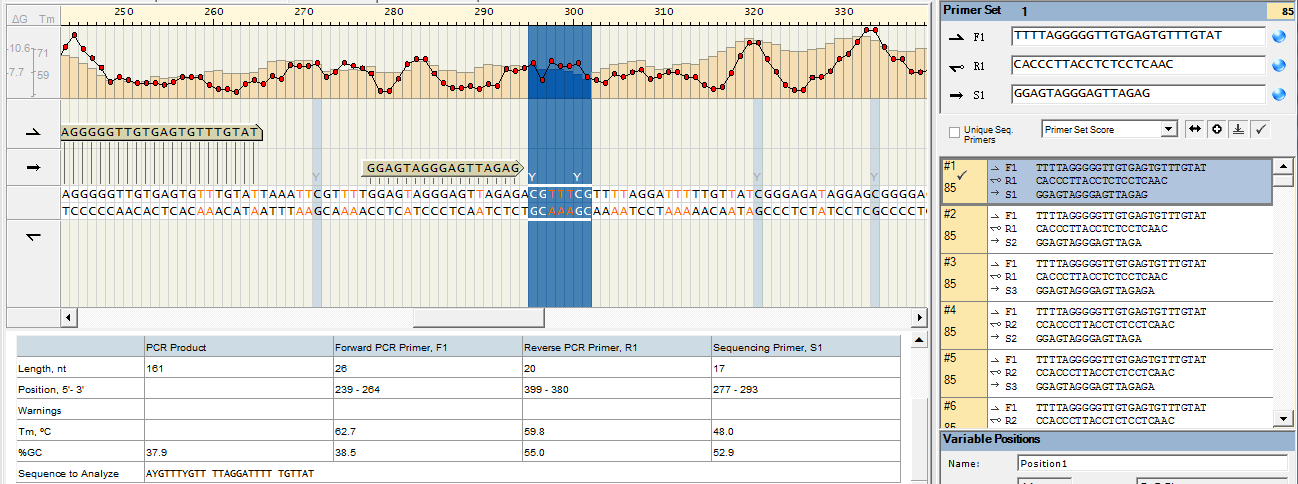


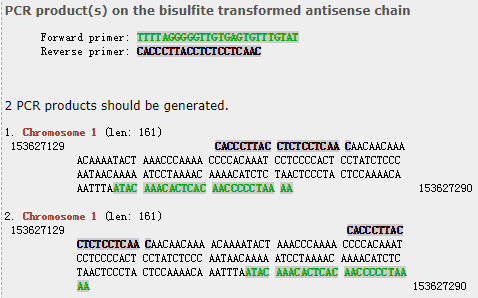


5 cg12667002

>hg19_wgEncodeHaibMethyl450Gm12878SitesRep1_cg12667002 range=chr13:114811803-114812803 5'pad=0 3'pad=0 strand=- repeatMasking=none

GCGTGTCCGTGAAGAGTGGCGTCGAGGACCTTTTCTTGGGCTTAGTGACAGCTCCATTCCTTCTTTAGTACATTGTTCAAATCTTTTGCCCTTTTAATTGGGTTGTTTTCTTGTTACTTTTTAAAGTTCCCTGTATATTCTGGATACGAGTCTATCACAAGTGTGCTTTGCAAATATTTTCTTCCAGTTTGTGGCTTGTATTTCATTTTCTTACCATTGGCTTTGAAGAACAGAAGTTTTTAATTTCGATGGTCCAGTTTATCACATTTTTTTCTTGTATGATTTGTACCTGTGGTGTCATGTCTAAGAAAGTCTTGCCTATTCCACGGTTGTGGCGGTTTTCTCCTGGAAAGCATGTGCTTTCAGCGTTCACATTTAGTTCCATGATCTGTTTCAAGCTAATTTTCACTTATAGCACAAAGAAAAGGGTCGGGATTCTGTTTTTTCCATGTAGACATCCCTTTGTTCCGGCACCCTTGGCTGAAAGACGACCCTTCCTCCGTTGATGACTTTGGCACATTTACAGCTTAGTTGGCTGCATTGTGTGGGTCTGTTTGTGGCCTCTGTATTCTCTTCCATCTGAGGTAGGAGGCGGGACTGGACTCTGAAGGCAGGGCTCGGACACCGAACCAAATTGAGGACTAGCTGAGACAGGGATGGGTGGAAGCAGCTTTCCATCGGACACGCCTGCCAGTGTACCATGTCACCTTACCATTGCCATGGCAACGCCTGGAGGTTACCACCCCTTTCCATAGGAACAACCCCGTGACTCGGAAATTACCACCCTTTTTCTAGAAATGTCTGCATAATCTTTTAATTGACATATAGTTAAAAGTGAGGATAAATAAGACTGCAGCAGTGCCCGGAGGTGCCACTCTCAACACCCTGCCTATGGGGAACCCTTCTCTGCAGGAACGGCTGAGACTGTCAACACCCCGCCCGTGGGGAGTCCTGCTGTGCAGGAGCAGTTTCAGAGCTGCCACTGCCGCCTCCATAAAACT

Reverse sequence design primer：（pos1）

| **Primer Set 1** | | | | **Score: 84 Quality: Medium** | | |  |
| --- | --- | --- | --- | --- | --- | --- | --- |
| **Primer** | **Id** | | **Sequence** | **Nt** | **Tm, ºC** | **%GC** |  |
| 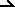PCR | F1 | | TTTTATTTTAGATGGAAGAGAATATAGAGG | 30 | 58.0 | 26.7 |  |
| 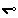PCR | R1 | | TTTCCATATAAACATCCCTTTATTCC | 26 | 57.2 | 30.8 |  |
| 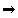Sequencing | S1 | | AGTTGTAAATGTGTTAAAGTTAT | 23 | 45.7 | 21.7 |  |
| Target Polymorphisms | Position9, Position10 | | | | | | |
| Sequence to Analyze | TAAYGGAGGA AGGGTYGTTT TTTAGTTAAG GGTGT | | | | | |  |
| Amplicon length | | 147 | | | | | |


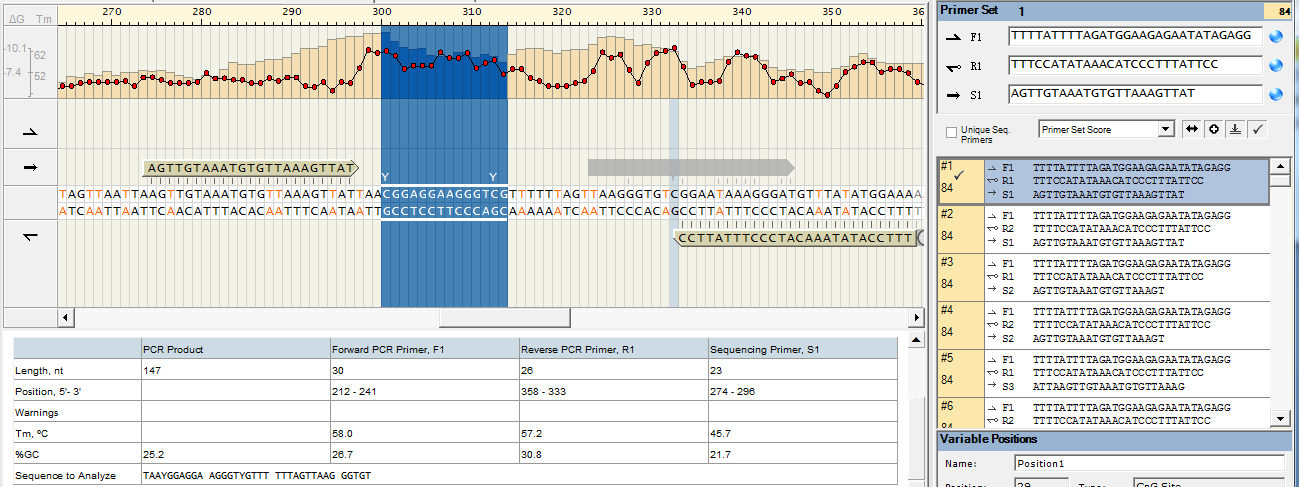

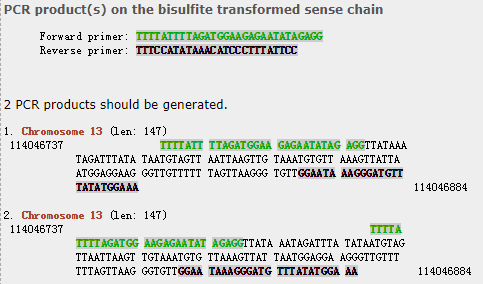

Supplement: Supplementary file 4 — Additional file 4:. Supplement 4. [file 13148_2020_871_MOESM4_ESM.docx]
